# Persistence of oxidant and protease burden in the airways after smoking cessation

**DOI:** 10.1186/1471-2466-9-25

**Published:** 2009-05-27

**Authors:** Noora Louhelainen, Paula Rytilä, Tari Haahtela, Vuokko L Kinnula, Ratko Djukanović

**Affiliations:** 1 Department of Medicine, Division of Allergy, University of Helsinki, Finland; 2 Department of Medicine, Division of Pulmonary Medicine, University of Helsinki, Finland; 3 Division of Infection, Inflammation and Repair, Southampton General Hospital, Southampton, UK

## Abstract

**Background:**

Oxidative stress is associated with the pathogenesis of cigarette smoke related lung diseases, but longitudinal effects of smoking cessation on oxidant markers in the airways are unknown.

**Methods:**

This study included 61 smokers; 21 with chronic bronchitis or COPD, 15 asthmatics and 25 asymptomatic smokers followed up for 3 months after smoking cessation. Fractional exhaled nitric oxide (FeNO), sputum neutrophil counts, sputum 8-isoprostane, nitrotyrosine and matrix metalloproteinase-8 (MMP-8) were investigated at baseline and 1 and 3 months after smoking cessation.

**Results:**

After 3 months 15 subjects had succeeded in quitting of smoking and in these subjects symptoms improved significantly. Unexpectedly, however, sputum neutrophils increased (p = 0.046) after smoking cessation in patients with chronic bronchitis/COPD. At baseline, the other markers did not differ between the three groups so these results were combined for further analysis. Sputum 8-isoprostane declined significantly during the follow-up at 3 months (p = 0.035), but levels still remained significantly higher than in non-smokers. The levels of FeNO, nitrotyrosine and MMP-8 did not change significantly during the 3 months after smoking cessation.

**Conclusion:**

Whilst symptoms improve after smoking cessation, the oxidant and protease burden in the airways continues for months.

## Background

COPD is related to smoking in most of the cases [1] and smoking cessation is the single most beneficial and cost-effective way to reduce COPD morbidity, hospital admissions[2] and COPD progression [1]. Given that as many as 30% of asthmatics smoke [3], smoking is now also seen as an important contributor to asthma pathogenesis. Thus, smoking asthmatics tend to have more severe disease than non-smoking asthmatics, their inflammatory features are different from those with typical asthma and their symptoms and inflammation is relatively resistant to corticosteroids [4]. Numerous cross-sectional studies have been conducted on current smokers, ex-smokers and COPD patients, while only a few longitudinal studies have assessed long-term effects of smoking cessation [5,6]. Smoking asthmatics have generally been excluded from most asthma studies.

Persistent inflammation in the airways of COPD patients may continue after quitting smoking. Thus a recent study, which analyzed pooled data from three bronchial biopsy studies, concluded that numbers of airway inflammatory cells, including CD4+ and CD8+ lymphocytes were largely similar in current smokers and ex-smokers [7]. Another longitudinal study showed persistence of raised sputum neutrophils and lymphocytes even one year after smoking cessation [6]. In contrast, asthmatics, who quit smoking for six weeks, showed reduced numbers of sputum neutrophils [5]. These studies suggest ongoing inflammation, at least in COPD patients, after smoking cessation.

Little is known about the effects of smoking cessation on any oxidant marker in the longitudinal setting. In previous cross-sectional studies [8,9], we have found significant increases of several oxidant markers (such as 8-isoprostane, inducible nitric oxide synthase and nitrotyrosine) in the sputum samples of asymptomatic smokers when compared to never smokers and also higher marker levels in COPD patients when compared with non-symptomatic smokers. We have also found that the levels of several markers of oxidative stress in induced sputum were very similar in never smokers and healthy ex-smokers who had quit smoking more than 20 years ago [8,9], suggesting that oxidative stress declines with time after stopping of smoking, although the speed at which these improvements take place has remained unknown.

In the current study we have investigated whether smoking cessation has rapid effects on sputum markers of oxidative/nitrosative stress in exhaled air and sputum of subjects with chronic bronchitis/COPD, asthma and asymptomatic smokers during a period of 3 months after quitting smoking. The chosen markers included 8-isoprostane, fractional nitric oxide (FeNO), and nitrotyrosine; to our knowledge, the effect of smoking cessation on these markers is unknown. Matrix metalloproteinases (MMPs) have been suggested to associate with the pathogenesis of COPD and asthma [10-12] and their complex activation can be triggered by increased oxidative stress [13,14]. The effects of smoking cessation on MMPs are also unknown. Based on our recent sputum studies of several MMPs in mild COPD [15], we selected the analysis of sputum MMP-8 for the current study.

## Methods

### Study design

This was a prospective study where subjects were recruited from three different smoking cessation clinics: Helsinki University Central Hospital, Southampton University Hospitals Trust (SUHT) and the "Quitters' specialist smoking cessation service" of the Southampton and South West Hampshire Region. Patients were examined prior to commencing the smoking cessation programme and the effects of smoking cessation on airway inflammation investigated in exhaled air and sputum samples at baseline and 1 and 3 months after successful cessation, which was confirmed by frequent exhaled carbon monoxide analyses. The study was approved by the Ethics Committees of Helsinki University Hospital and the Southampton University Hospital. All subjects gave full informed consent.

### Subjects

Subjects were classified into three categories (Table 1): 1) smokers with cough and sputum production but normal spirometry (Stage 0 COPD in earlier GOLD classification), referred to as chronic bronchitis (n = 7) in this study, and smokers with varying severity of COPD (n = 14), all having FEV/FVC<70%), 2) smoking asthmatics and 3) asymptomatic smoking subjects with normal lung function (FEV/FVC>70%). None of the subjects in the first and third groups were atopic according to skin prick tests or had significant (>12%) bronchodilator reversibility. Two of smokers with bronchitis/COPD were treated with inhaled steroids (average dose 1200 μg/day) whereas all asthmatic were treated with inhaled steroid (average dose 680 μg/day). The dose of inhaled steroids was kept stable during the entire study.

**Table 1:** Subject characteristics

	**Non symptomatic**	**Bronchitis/COPD**	**Asthma**
**Number**	25	21	15
**Age, yr**	41 (18–64)	56 (41–72)	42 (18–58)
**Sex M/F**	11/14	7/14	5/10
**FEV1 (%pred)**	97,8 (86–126)	76,8 (25–109)	95,6 (61–127)
**FVC (%pred)**	98,6 (40–124)	99 (50–203)	100,5 (36–139)
**FEV1/FVC ratio%**	82,6 (71–109)	66,7 (28–87)	76,6 (74–79)
**DLCO/VA**	87 (50–118)	53,3 (28–77)	85,4 (57–107)
**Pack years**	22 (4,5–60)	39 (17–75)	22 (6–48)

Of the total of 61 subjects enrolled, only 6 smokers with bronchitis/COPD, 6 subjects with asthma and 3 asymptomatic smokers managed to quit smoking for 3 months. Subjects who had not been successful at stopping smoking also failed to keep follow-up appointments, leaving the 15 subjects. The final analyses were conducted on the quitters who also had produced good quality sputum specimens. The number of the analyses was lower than the total number of subjects who succeeded to quit smoking due to unrepresentative sputum specimens during the control visit. Respiratory symptoms and health status in subjects were assessed with St Georges Respiratory Questionnaire (SGRQ) at baseline and at 1 and 3 months. Flow-volume spirometry and diffusion capacity measurement were conducted using standard methods [16].

### Sputum induction and processing

Sputum induction was conducted in all centres using the guidelines of the European Respiratory Society's Task Force [17]; the same standard operation protocol has been described in previous studies of our laboratory [8,9]. One author (PR) participated to the processing in each centre. Sputum was induced with 4.5% hypertonic saline given at 5-min intervals for a maximum of 20 min, with mean induction times being similar in the three subject groups. The mucoid components of sputum were selected in order to reduce salivary contamination and processed as described [18] with four volumes of dithioerythritol (DTE). DTE improves cell and mediator recovery without causing cell activation [19]. The suspensions were filtered through 70-μm nylon gauze and centrifuged at 400 g at 4°C for 10 min. The DTE-processed samples were used to make cytospins (450 rpm for 6 min) for total and differential cell counts, 400 non-squamous cells were calculated. The slides were frozen at -20°C until analysis. The supernatant was frozen at -80°C for biochemical analysis and immunoassay.

### 8-isoprostane analysis

8-Isoprostane (8-iso-PGF2 α) concentrations in induced sputum samples were determined by specific enzyme immunoassay (EIA) kit (Cayman Chemical, Ann Arbor, MI, USA) with standard curves using purified 8-isoprostane. The sputum samples were diluted in the EIA buffer provided by the manufacturer and analysed as recently described and tested by our group [8]. Values were expressed as pg/ml.

### Exhaled NO measurement

The FeNO measurements (Niox; Aerocrine AB, Sweden) were performed according to the ATS guidelines [20]. Expiratory airflow was 50 ml/s against a flow resistor, with an exhalation time of 10 sec. The mean value from a 3-second period from the end-exhaled NO plateau was recorded. At least three successive FeNO measurements were performed and the mean value was used for analysis.

### Immunocytochemical analysis of sputum cells

Nitrotyrosine (NT) was assessed by immunocytochemistry by counting the NT-positive cells from amongst a total of 400 cells counted in every cytospin. Polyclonal nitrotyrosine antibody (Upstate Lake Placid, NY, US) in 1:100 dilution and the Zymed Broad spectrum antibody (Zymed Laboratories Inc., South San Francisco, CA, USA) as the secondary antibody were used as described by Rytilä et al [9]. The Zymed ABC Histostain-Plus Kit (Zymed Laboratories Inc.) was used according to the manufacturer's protocol to identify positively stained cells.

### Measurement of MMP-8

MMP-8 was determined by commercially available ELISA kit (Amersham Biosciences, Cardiff, UK) according to the manufacturer's instructions.

### Statistical analysis

All statistical analyses were performed using the SPSS 10.0 software program (SPSS Inc., Chicago, IL). As the data were not normally distributed, non-parametric tests were used for all comparisons. Data for individual variables from the several groups were first analyzed by the Kruskal-Wallis test followed by the Mann-Whitney U-test. All differences within sets of paired data were analysed by the non-parametric Wilcoxon signed rank test. We accepted p values of <0.05 as significant.

## Results

**Figure 1:**
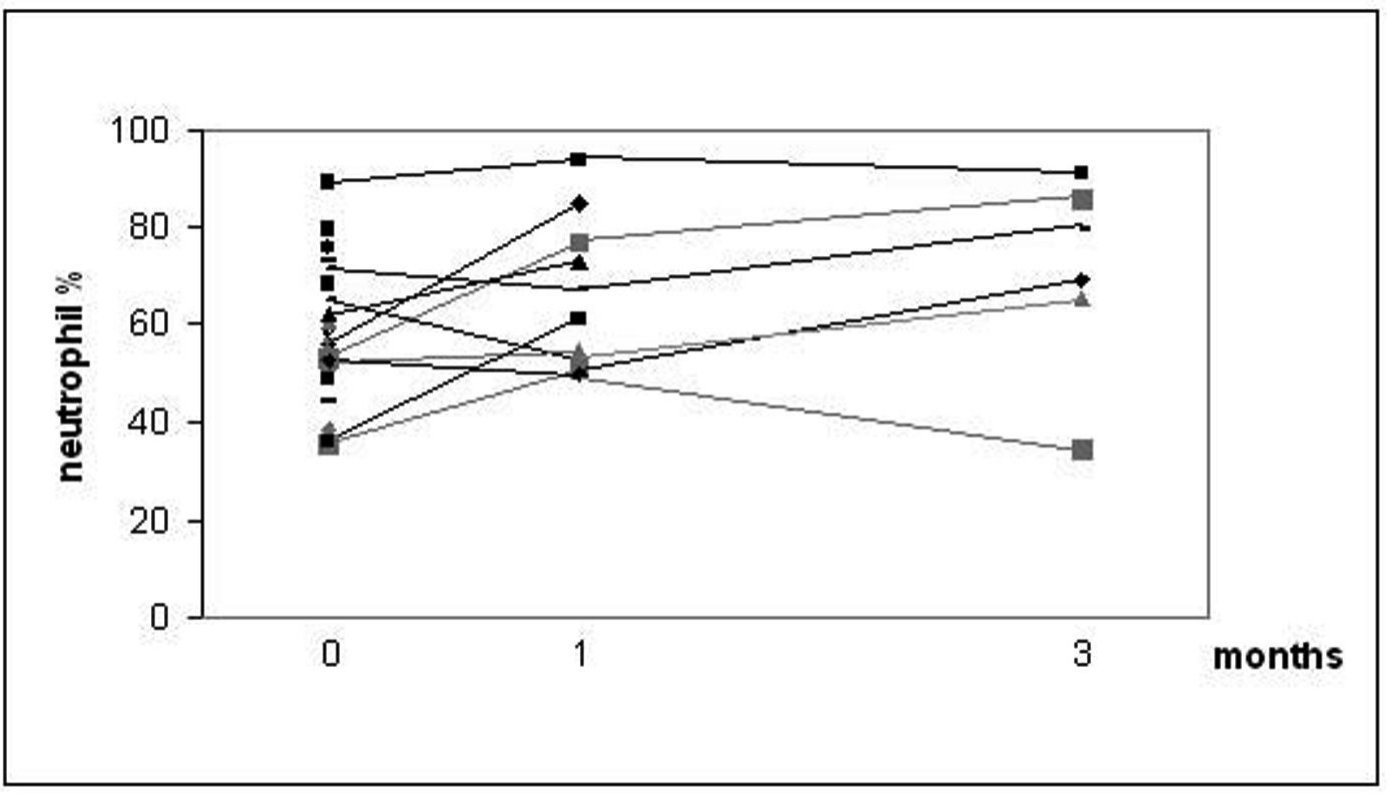
**Sputum neutrophils (%) in the induced sputum of the subjects with bronchitis and COPD**. P-value was calculated with Wilcoxon signed rank test. P = 0.046.

Spirometry values (FVC, FEV1, FEV/FVC) did not change significantly after smoking cessation when compared to baseline in any of the groups, reasons include the short follow-up and the fact that most participants were "healthy smokers", had chronic bronchitis/COPD or mild asthma. In the group of chronic bronchitis/COPD the percentage of sputum neutrophils increased significantly after 3 months of smoking cessation (p = 0.046) (Fig 1), while the cell counts remained similar in the asymptomatic (healthy) smokers and the asthmatics.

**Figure 2:**
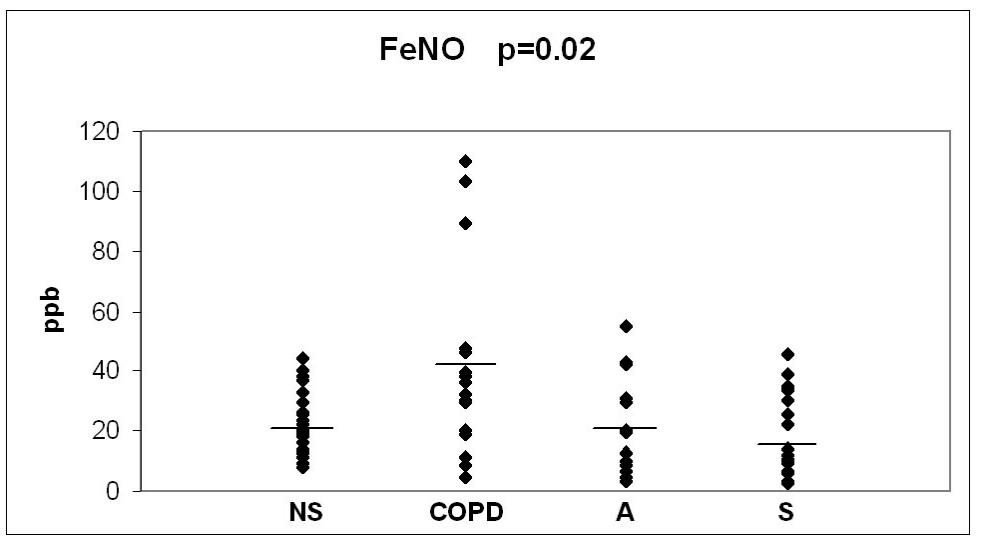
**Fractional exhaled nitric oxide (FeNO) in bronchitis and COPD patients (COPD), asthmatics (A) and asymptomatic smokers (S) at the beginning of the study**. The values of non smokers (NS) have been gathered from previously published materials where non-smokers were combined from two groups of subjects i.e. never smokers and ex smokers who had quitted smoking over 20 years ago. The levels of each marker in these two groups of non-smokers have been found to be very similar [8,9,15,20]. P-value was calculated with Kruskall-Wallis test.

**Figure 3:**
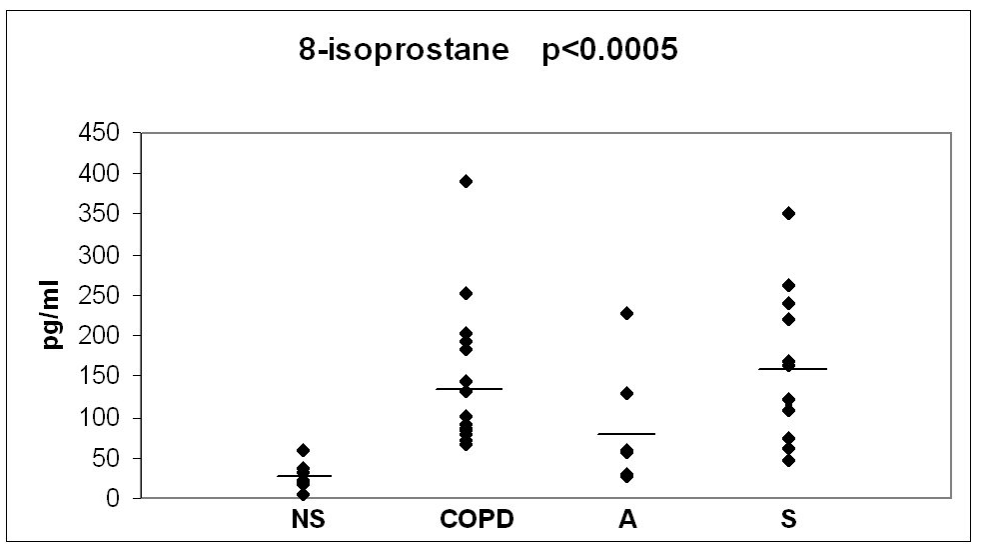
**Levels of sputum 8-isoprostane in bronchitis and COPD patients (COPD), asthmatics (A) and asymptomatic smokers (S) at the beginning of the study**. The values of non smokers (NS) have been gathered from previously published materials where non smokers were combined from two groups of subjects i.e. never smokers and ex smokers who had quitted smoking over 20 years ago. The levels of each marker in these two groups of non-smokers have been found to be very similar [8,9,15,20]. P-value was calculated with Kruskall-Wallis test.

**Figure 4:**
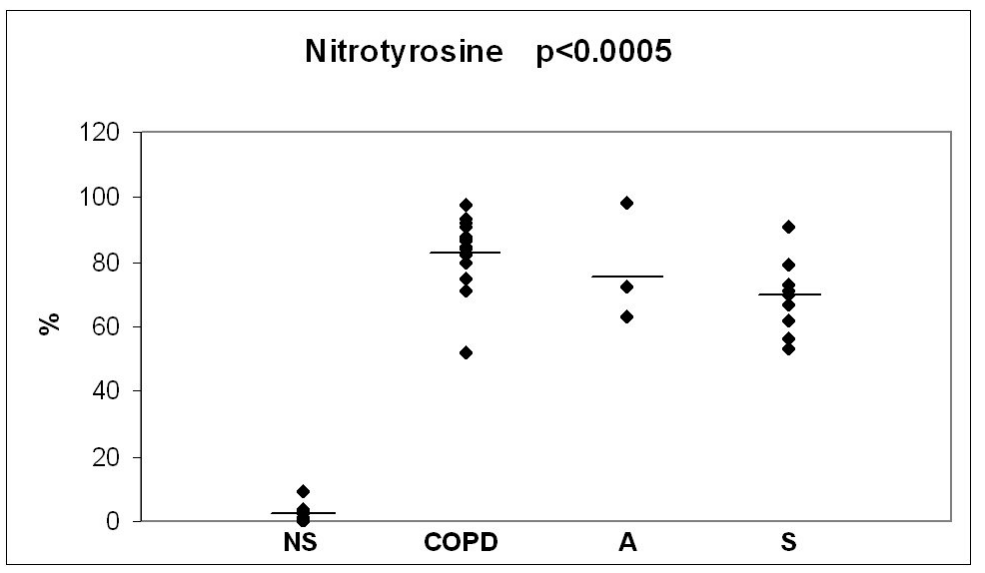
**Levels of sputum nitrotyrosine (% of positive cells) in bronchitis and COPD patients (COPD), asthmatics (A) and asymptomatic smokers (S) at the beginning of the study**. The values of non smokers (NS) have been gathered from previously published materials where non-smokers were combined from two groups of subjects i.e. never smokers and ex smokers who had quitted smoking over 20 years ago. The levels of each marker in these two groups of non-smokers have been found to be very similar [8,9,15,20]. P-value was calculated with Kruskall-Wallis test.

**Figure 5:**
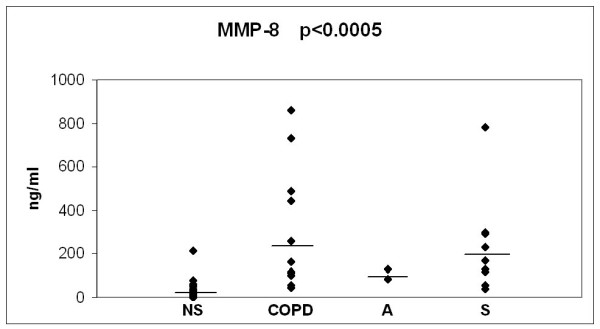
**Levels of sputum MMP-8 in bronchitis and COPD patients (COPD), asthmatics (A) and asymptomatic smokers (S) at the beginning of the study**. The values of non smokers (NS) have been gathered from previously published materials where non smokers were combined from two groups of subjects i.e. never smokers and ex smokers who had quitted smoking over 20 years ago. The levels of each marker in these two groups of non-smokers have been found to be very similar [8,9,15,20]. P-value was calculated with Kruskall-Wallis test.

The baseline values of the 3 oxidant markers and MMP-8, investigated in the 3 study groups, in comparison to the values in non-smokers/ex smokers (over 20 years after smoking cessation) are presented in Figs 2, 3, 4 and 5. The values of non-smokers have been published in the recent studies of our laboratory with similar sputum processing and analyses [8,9,15,21,22] and contain pooled data both from never smokers (n = 20) and ex-smokers who had stopped >20 years ago (n = 12); altogether six control specimens were re-analyzed for the current study showing very similar levels as in those earlier investigations. As shown in Figs 2, 3, 4 and 5, the levels of all markers were significantly lower (p < 0.001 for each marker) in non-smokers than in smokers. However, the markers did not differ significantly between the asymptomatic smokers, smoking asthmatics or smokers with chronic bronchitis/COPD.

**Figure 6:**
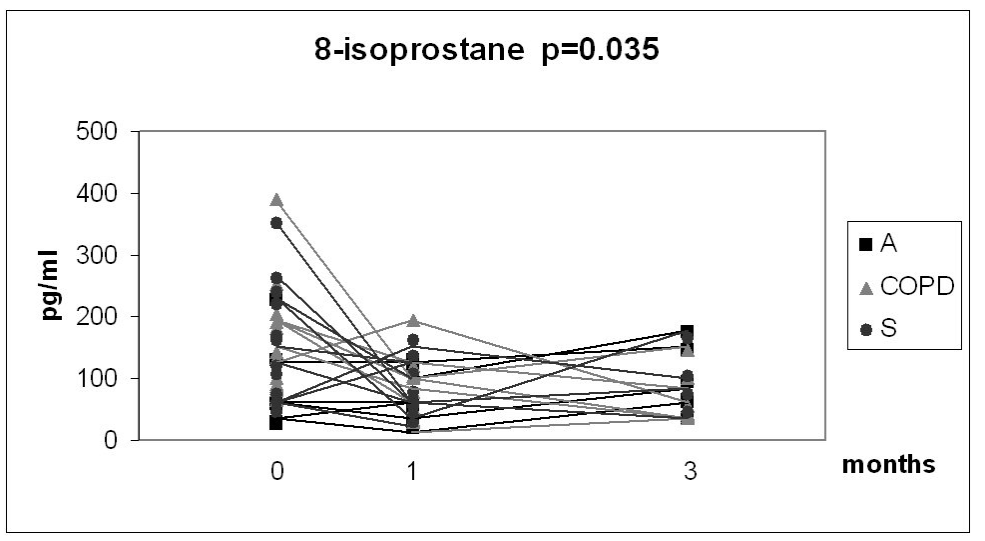
**Levels of sputum 8-isoprostane in the combined groups of chronic bronchitis and COPD (COPD), asthma (A) and asymptomatic smokers (S)**. Individual subgroups are presented as corresponding symbols and shown in the panels. Only 8-isoprostane in the combined group showed a significant decline (p = 0.035) after smoking cessation calculated with Wilcoxon signed rank test. The number of the analyses was lower than the total number of subjects who succeeded in smoking cessation due to unrepresentative sputum specimen during the control visit.

**Figure 7:**
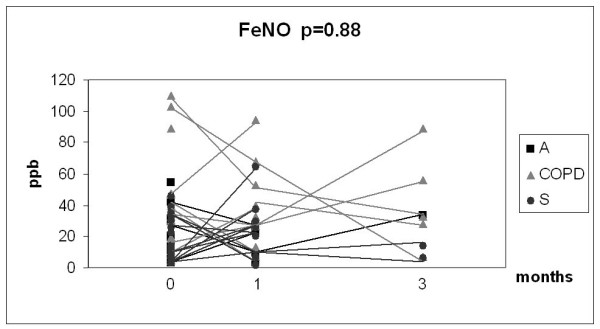
**Exhaled fractional nitric oxide (FeNO) in the combined groups of chronic bronchitis and COPD (COPD), asthma (A) and asymptomatic smokers (S)**. Individual subgroups are presented as corresponding symbols and shown in the panels. Only 8-isoprostane in the combined group showed a significant decline (p = 0.035) after smoking cessation calculated with Wilcoxon signed rank test. The number of the analyses was lower than the total number of subjects who succeeded in smoking cessation due to unrepresentative sputum specimen during the control visit.

**Figure 8:**
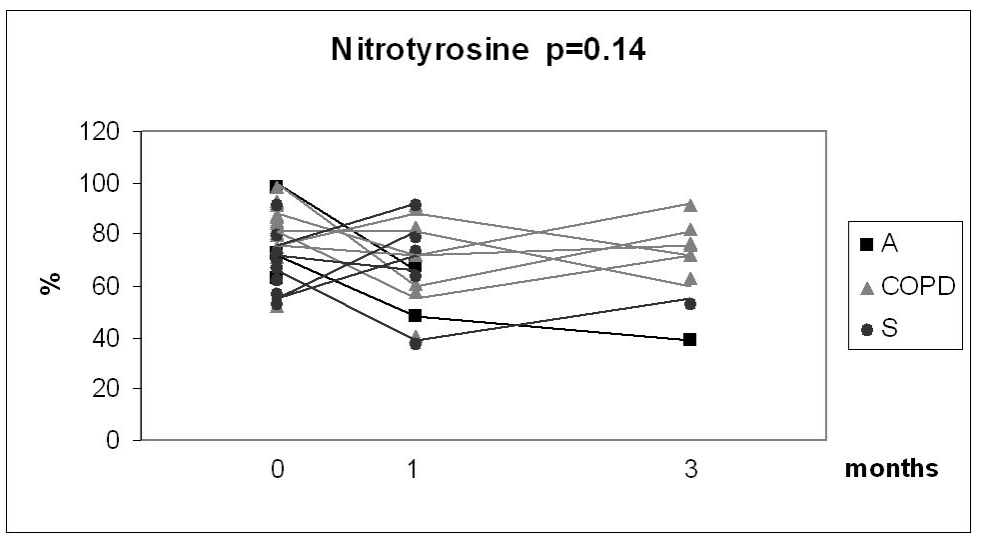
**Percentage of nitrotyrosine positive cells in the combined groups of chronic bronchitis and COPD (COPD), asthma (A) and asymptomatic smokers (S)**. Individual subgroups are presented as corresponding symbols and shown in the panels. Only 8-isoprostane in the combined group showed a significant decline (p = 0.035) after smoking cessation calculated with Wilcoxon signed rank test. The number of the analyses was lower than the total number of subjects who succeeded in smoking cessation due to unrepresentative sputum specimen during the control visit.

**Figure 9:**
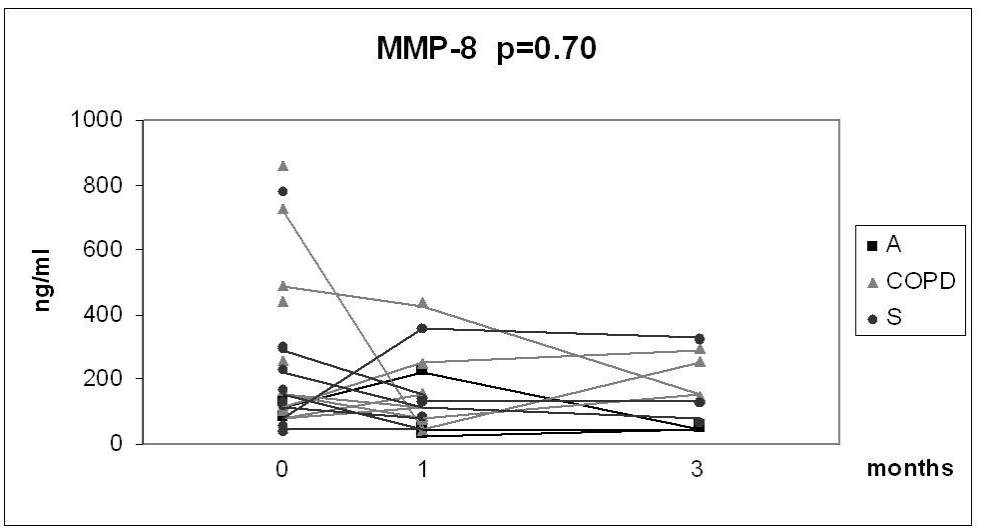
**Levels of sputum MMP-8 in the combined groups of chronic bronchitis and COPD (COPD), asthma (A) and asymptomatic smokers (S)**. Individual subgroups are presented as corresponding symbols and shown in the panels. Only 8-isoprostane in the combined group showed a significant decline (p = 0.035) after smoking cessation calculated with Wilcoxon signed rank test. The number of the analyses was lower than the total number of subjects who succeeded in smoking cessation due to unrepresentative sputum specimen during the control visit.

Given the relatively small number of cases in each group and the overlap between the values, the results of the 3 month longitudinal study were combined into one analysis as presented in Fig 6, 7, 8 and 9. In this combined group, only sputum 8-isoprostane changed significantly during the follow-up at 3 months (p = 0.035). The change of isoprostane at one month was not significant although a trend to decrease could be detected (p = 0.07). The levels of 8-isoprostane remained higher than in non-smokers after 3 months of smoking cessation (see Fig 3). FeNO and nitrotyrosine positive sputum cells showed no tendency to change. There was a trend for MMP-8 to decline after smoking cessation, but the levels also remained higher than in non-smokers (see Fig 5).

Symptoms assessed by the St Georges Respiratory Questionnaire (SGRQ) improved significantly (over four points based on the SGRQ manual) in those subjects who quitted smoking, especially at the one month visit but were still lower at three months when compared to the beginning of the study. Improvement was seen in all parts of the questionnaire: symptoms, activity and impact scores (Table 2).

**Table 2:** Symptoms score

**Visit**	**Symptoms score**	**Activity score**	**Impact score**	**Total score**
**0**	47 (0–91)	39 (0–100)	19 (0–69)	30 (2–82)

**1**	16 (0–53)	18 (0–61)	4 (0–38)	10 (0–41)

**3**	26 (0–77)	18 (0–55)	14 (0–43)	21 (0–61)

Symptoms score is concerned with the effect of respiratory symptoms, their frequency and severity, activity score activities that cause or are limited by breathlessness and impacts score covers a range of aspects such as social functioning and psychological disturbances resulting from airways disease. Total score summarises the impact of the disease on overall health status. According to SGRQ manual, decrease of more than four points in the questionnaire is clinically significant. SGRQ total score and subdomains scores are given only for the quitters.

## Discussion

Smoking cessation still remains the best therapeutic intervention in smokers, especially in individuals who have evidence of COPD or asthma [23]. Despite major efforts in developing cessation programs and drugs to ease the process, success rates are generally low to moderate [24-26]. In our study 25% of the smokers succeeded in quitting of smoking at least for the time of the follow-up, a success rate which is good and close to the published results of programs involving COPD patients who had received active counseling [27]. Understanding the mechanisms whereby quitting smoking leads to better health is important not only for a better understanding of disease mechanisms but also to obtain relevant information which can be presented to patients in order to encourage them not to go back to smoking which, according to current evidence, happens to a large proportion of patients.

Our study shows that oxidative stress can persist even three months after patients stop smoking. Contrary to what was anticipated, but in agreement with previous results, neutrophil counts increased in COPD after smoking cessation [6]. Published evidence suggests that inflammation continues after smoking cessation, although there have been no thorough longitudinal studies of how long inflammation persists and whether it is definitely irreversible. Cross-sectional studies have shown elevated inflammatory indices in terms of macrophages, neutrophils and eosinophils in ex-smokers when compared to never smokers [28-31], and a shift from CD4+ (T-helper) to CD8+ (T-suppressor) predominance in heavy smokers and COPD [7,32]. There have been only few prospective longitudinal studies comparing indices of inflammation before and after smoking cessation [5,6], but no studies have been conducted on markers of oxidative/nitrosative stress after smoking cessation. Our results confirm previous findings that airway inflammation persists after smoking cessation for months. Whilst this study shows that there is some decline in oxidative stress (as suggested by the decline in 8-isoprostane), the ongoing oxidant and protease load is seen months after smoking cessation. Given that COPD is also systemic disease further studies are needed to assess how the biomarkers that reflect systemic oxidative stress/inflammation will be changed after smoking cessation. Importantly, symptoms declined significantly already one month after patients stopped smoking underlining the complexity of multiple consequences related to smoking cessation.

Studies on asthma have to date concentrated on non-smoking subjects in order to avoid the known effects of smoking confounding the pathogenic mechanisms under investigation. Cigarette smoking in adults with established asthma is associated with increased symptom severity and exacerbation frequency [4], accelerated decline in lung function [33] and impaired response to inhaled and oral corticosteroids [34-36], features which are associated with COPD. In addition to having eosinophilic airway inflammation normally observed in asthma, asthmatic smokers have neutrophilic inflammation [37], a further feature that is typical of COPD. However, there is limited knowledge of the effects of smoking on asthmatic airway inflammation. In the study of Chaudhuri et al. [5] sputum neutrophil counts decreased after 6 weeks of smoking cessation, which is in contrast with our observation as we saw no significant changes in neutrophils during the three months after smoking cessation in the asthmatics. Although the number of asthmatics was low, the results exclude dramatic improvements in airway inflammation after smoking cessation in asthma.

Looking at the bronchitis/COPD group alone, the percentage of sputum neutrophils increased significantly after 3 months of smoking cessation in bronchitis/COPD. Whilst these results have to be interpreted with caution because of low subject numbers, together with results from a previous study of 12 COPD patients and 16 asymptomatic smokers [6], which showed high neutrophil counts one year after smoking cessation, this would suggest that neutrophilic inflammation does not subside long after subjects stop smoking.

8-isoprostane was studied as a potential marker of ongoing oxidative stress in lung diseases [38] that is reliably detected in *in vivo *specimens [39]. It has been suggested to be a specific marker for lipid peroxidation [40] and is sensitive in evaluating oxidative/nitrosative stress in the airways. Furthermore, it is stable, allowing determination in frozen specimens [38]. Increased levels of 8-isoprostane have been previously measured in sputum samples of stable COPD [8] and mild/moderate/severe asthma when compared to healthy controls [21,41], although levels have been highly variable. 8-isoprostane has also been detected and shown to be elevated in the exhaled breath condensate in many lung diseases [42-45] and confirmed by reference analytical techniques by Montuschi and co-workers [46].

Smoking alone increases levels of sputum 8-isoprostane when compared to non-smoking controls [8]. In the present study the baseline levels of 8-isoprostane were already highly variable especially in the asthmatics. When the results from three groups were combined the levels of 8-isoprostane in the individual subgroups declined significantly after smoking cessation, but remained higher than in non-smokers (Figs 2, 3, 4, 5, 6, 7, 8 and 9). Overall, the present results suggest that there is clear trend but no major or immediate decline in the oxidative stress within the first months after smoking cessation when evaluated by sputum 8-isoprostane.

Fractional nitric oxide (FeNO) is elevated in inflammatory diseases such as asthma [47], especially in atopic asthma and the elevation is generally limited to steroid-naïve asthmatics. It is one of the few non-invasive markers that have been used in the clinical assessment of asthmatic patients [48]. FeNO is decreased by cigarette smoke but most studies suggest that FeNO is not significantly different from normal in stable COPD [9,49-51]. To our knowledge, FeNO has not been investigated in longitudinal studies after smoking cessation. Based on past investigations which have shown FeNO to decrease in smokers [9,52], it can be speculated that FeNO might increase after smoking cessation. Our recent cross-sectional study showed levels of FeNO to be approximately 11 pbb in healthy smokers and 22 pbb in non-smokers [9]. In the present study FeNO was highly variable in asthma, possible reasons being not only the extent of smoking but also variable severity and usage of inhaled corticosteroids; each asthmatic was on inhaled steroids, which were not changed during the course of this study. FeNO appeared to be moderately variable in bronchitis/COPD but in both groups it remained unchanged after smoking cessation. Exact comparisons are difficult because all patients with asthma in this study were on inhaled steroids, whereas most of the patients with COPD were not. Overall, unchanged FeNO levels suggest that oxidative/nitrosative stress does not change markedly during 3 months after smoking cessation. Moreover, the regulation of FeNO is complex, and many reactions associated with its changes in smoking asthmatics and COPD patients are still incompletely understood and difficult to interpret in real life.

The final marker of oxidative stress investigated was nitrotyrosine which has been suggested to play a major role in the pathogenesis of airway remodelling [53]. Numerous nitrotyrosine positive sputum cells have been seen not only in COPD [54] but also in current cigarette smokers without airway obstruction [9]. Nitrotyrosine positive sputum cell counts are elevated in healthy smokers when compared to never smokers [9], with further increases in COPD [54]. The numbers of nitrotyrosine positive cells in the sputum remained unchanged after smoking cessation for 3 months, which is in agreement with the results obtained with other markers of oxidative/nitrosative stress. It is apparent that smoking cessation does not lead to any immediate changes in the oxidant burden in asthma or COPD. This may be associated with the persistence of ongoing inflammation and possibly also with the activation status of the inflammatory cells.

Several MMPs including MMP-8, MMP-9 and MMP-12 have been associated with COPD [10]; in our recent study only the levels of MMP-8 were higher in chronic bronchitis compared to asymptomatic smokers [15]. Since oxidative stress and cigarette smoke associated oxidants may also enhance MMP activation, it can be speculated that the levels of MMPs might decrease after smoking cessation. In the present study MMP-8 levels showed some tendency to decrease but there was a high individual variation in the MMP-8 levels after 3 months quitting of smoking. The levels of MMP-8 in bronchitis/COPD after stopping of smoking at 3 months were still much higher than in non-smokers as measured in our recent study [15] (see also Fig 2, 3, 4 and 5). These results suggest persistence of the protease cascade imbalance months after smoking cessation.

The current study has a number of limitations; the most important being is the modest success of the smoking cessation and the short follow-up. The success rate of smoking cessation was also very different between the groups, which may be explained by differences in smoking cessation programs, including clinical care setting, the use of smoking cessation aids e.g drugs and the intensity of counselling. Another limitation is the variability of the subject characteristics and medications within the groups. COPD group also included subjects with symptoms of chronic bronchitis. The use of steroids differed between the groups, though the use in individual subjects was the same at the baseline and in the control visit. However, oxidant markers varied even more inside the asthma group than in the bronchitis/COPD group. These realities reduce the statistical power of our analyses. The variability in inflammatory/oxidant markers in sputum specimens was also reported in a recent study of Sapey et al who examined several cytokines and oxidants in spontaneous sputum samples from patients with COPD; that particular study revealed high cytokine variability in consequent sputum specimens even in stable COPD [55].

One final important finding of the present study is the decline in symptoms despite persistent neutrophilic airway inflammation and oxidative stress. Symptoms already reduced one month after smoking cessation, when none of the other markers had yet declined significantly. This finding is important and suggests that clinical improvement does not necessarily correlate with objective assessment of asthma/COPD or that these biomarkers may not be the best ones in regard to clinical relevance in COPD and/or that the mechanisms of COPD are still poorly known. Such discrepancies have, however, been found in many clinical trials where symptom score but not objective monitoring of the disease reveals significant improvement within the first months of treatment. Whilst our study suggests that smoking cessation overall does not cause any major immediate decline in the oxidant burden in COPD or asthma, the wide individual variability in the outcomes measured raises the questions as to whether some of these markers may be predictive of which patient goes on to develop further lung damage and in which patient the disease processes may be arrested. It is possible that the participants' assessment of their symptoms was influenced by their positive perception of successful cessation of smoking and further studies in which this confounding factor is controlled for are needed. Further studies are also needed to investigate the specific pathways that may remain activated after smoking cessation and the development of new antioxidant/redox modulatory and/or protease inhibitor strategies directed to these pathways also after smoking cessation.

## Conclusion

There are only a few studies about the effects of smoking cessation on oxidant markers in the longitudinal setting. We investigated whether smoking cessation has effects on sputum markers of oxidative/nitrosative stress in exhaled air and sputum of subjects with chronic bronchitis/COPD, asthma and asymptomatic smokers during a period of 3 months after quitting smoking. Sputum neutrophils increased after smoking cessation in patients with chronic bronchitis/COPD, but the levels of FeNO, nitrotyrosine and MMP-8 did not change significantly during the 3 months after smoking cessation in any of the groups. Symptoms decreased significantly in those subjects who quitted smoking. We conclude that although symptoms improve after smoking cessation, the oxidant and protease burden in the airways continues at least for months.

## Competing interests

The authors declare that they have no competing interests.

## Authors' contributions

NL performed part of the statistical analysis, created the figures and drafted the manuscript. PR participated in the selection of patient material, manuscript writing and helped with the statistics. TH, VLK and RD participated in the design and coordination of the study, collection of patient material and writing of the manuscript. All authors have read and approved the final manuscript.

## Pre-publication history

The pre-publication history for this paper can be accessed here:


